# Tailoring of the chlorine sensing properties of substituted metal phthalocyanines non-covalently anchored on single-walled carbon nanotubes

**DOI:** 10.1039/c8ra05529g

**Published:** 2018-09-21

**Authors:** Anshul Kumar Sharma, Aman Mahajan, Subodh Kumar, A. K. Debnath, D. K. Aswal

**Affiliations:** Material Science Laboratory, Department of Physics, Guru Nanak Dev University Amritsar 143005 India aman.phy@gndu.ac.in; Department of Chemistry, Guru Nanak Dev University Amritsar 143005 India; Technical Physics Division, Bhabha Atomic Research Centre Mumbai 400085 India; CSIR-National Physical Laboratory New Delhi 110012 India

## Abstract

To investigate how central metal tunes the synergetic interactions between substituted metallo-phthalocyanine and single-walled carbon nanotubes in enhancing the gas sensing properties, a comparative study has been performed by varying the central metal ion in fluorinated metal phthalocyanines and single-walled carbon nanotube hybrid. Hybrids of metal(ii)-1,2,3,4,8,9,10,11,15,16,17,18-24,25-hexa-decafluoro-29*H*,31*H*-phthalocyanine/single-walled carbon nanotube (F_16_MPc/SWCNTs–COOH, where M = Co, Zn) have been synthesized through π–π stacking interactions using the solution route. Spectroscopic (FT-IR, UV-vis, XPS and Raman), electron microscopic (TEM and FE-SEM) and TGA investigations have confirmed the successful functionalization and interaction of SWCNTs–COOH with F_16_MPc. Parts per billion (ppb) level Cl_2_-selective chemiresistive gas sensors have been fabricated on glass substrates with precoated gold electrodes by using these hybrids. The responses of various F_16_MPc/SWCNTs–COOH sensors have demonstrated the central metal ion-dependence in the sensitivity of Cl_2_.

## Abstract 2

Schematic view of the interaction between Cl_2_ and S_1_/S_2_ hybrid sensor.

## Graphical abstract

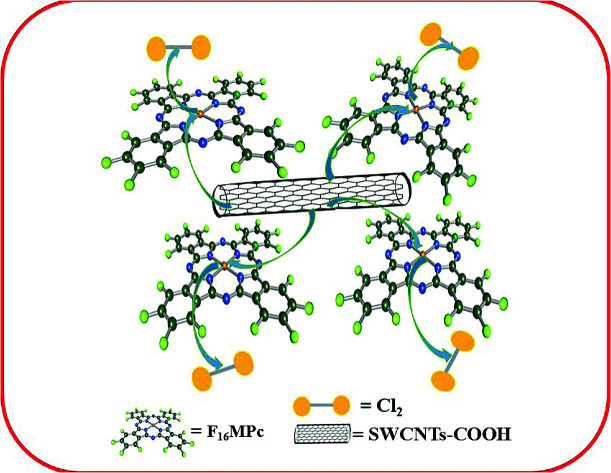

## 1. Introduction

Chlorine (Cl_2_) is commonly used in water purification, pharmaceuticals, textiles, plastics, agrochemicals and household cleaning products *etc.* Despite being a toxic gas with an occupational exposure limit (OEL) of 500 ppb for a time-weighted average of over eight hours, it can cause distress in the respiratory system and severely affect the environment and mankind.^1,2^ The very precise monitoring of Cl_2_ at the parts per billion (ppb) or parts per trillion (ppt) level has led to the development of economical, flexible, compact and low power consuming sensors. Different materials like metal oxides,^3–5^ organic semiconductors^6,7^ and carbon-based nanomaterials^8,9^ have been widely explored for the fabrication of gas sensors. Metal oxide-based (particularly SnO_2_, ZnO, WO_3_, TiO_2_, V_2_O_5_) chemiresistive gas sensors have been investigated for the detection of various toxic gases.^10,11^ In organic semiconductors, polycyclic aromatic hydrocarbons, phthalocyanines, porphyrin derivatives and polymers have been used as excellent sensing materials for the detection of various harmful gases.^12,13^ Carbon-based nanomaterials, fullerenes, graphene, and carbon nanotubes (CNTs), have been demonstrated as promising gas sensing materials.^14,15^ However, the gas sensing characteristics of CNT hybrids with noble metal nanoparticles,^16^ metal oxides^17^ and organic semiconductors^18,19^ are better in comparison to pristine CNTs due to the better charge transfer between the hybrid and gas analytes.

Among the organic semiconductors, metallo-phthalocyanines (MPcs) are attractive choices for the noncovalent functionalization of CNTs because of the synergic interaction of MPcs with CNTs due to π–π interactions.^20,21^ MPcs have emerged as outstanding sensing materials in highly selective, sensitive and reversible chemiresistive gas sensors to detect various toxic gases due to their conjugated macrocyclic units.^22,23^ We have previously reported the nanostructured growth of substituted MPcs for ppb level Cl_2_ gas sensors with detection limits as low as 5 ppb.^24,25^ Monllau *et al.*^26^ reported highly sensitive multiwalled carbon nanotubes and epoxy resin-based amperometric sensors for the detection of free chlorine in water, at concentrations as low as 20 μg L^−1^. Wang *et al.*^27^ fabricated lead phthalocyanine modified CNTs with enhanced NH_3_ sensing performance as compared to pristine CNTs. Liang *et al.*^20^ developed substituted metal(ii) phthalocyanine/multi-walled carbon nanotube hybrid (TFPMPc/MWCNT, M = Co, Zn, Cu, Pb, Pd, and Ni) sensors where the central metal atoms play an important role in the high sensitivity and selectivity of the sensor towards NH_3_. The response of the TFPMPc/MWCNT hybrid sensor to ammonia vapour is in the order of Co > Zn > Cu > Pb > Pd ∼ Ni, which has been attributed to the binding energies of the MPc-NH_3_ system.^20^ Recently, we fabricated Cl_2_ sensors using hybrids of carboxylic functionalized multi-walled carbon nanotubes with hexadecafluorinated metal phthalocyanines, (F_16_MPc, M = Cu, Zn, Co) and the response of the sensors to Cl_2_ lies in the order of Co > Cu > Zn.^28–30^ Kaya *et al.*^31^ have shown that the response of the sensor to ammonia vapor in the concentration range 20–50 ppm is of the order CuPc-py > CoPc-py > H_2_Pc-py. It is worth mentioning that single-walled carbon nanotubes (SWCNTs) have certain superior features compared to the MWCNTs due to the comparatively smaller size, stronger inter-tube attraction, and larger specific area of SWCNTs that will enhance the gas adsorption capability and will enhance the gas sensing parameters of the sensor. In our previous study, we have shown that the F_16_CuPc/SWCNTs–COOH hybrid sensor seems to be a significantly better candidate for gas sensing applications in comparison to the F_16_CuPc/MWCNTs–COOH hybrid sensor.^28^

Taking these facts into consideration, in order to tune the Cl_2_ sensing properties of the MPc/CNTs hybrid and to verify the effect of the central metal in the phthalocyanine molecule, we have synthesized hybrids of SWCNTs–COOH with metal(ii)1,2,3,4,8,9,10,11,15,16,17,18-24,25-hexadecafluoro-29*H*,31*H*-phthalocyanine (F_16_MPcs, where M = Co, Zn).^32–34^

**Fig. 1:**
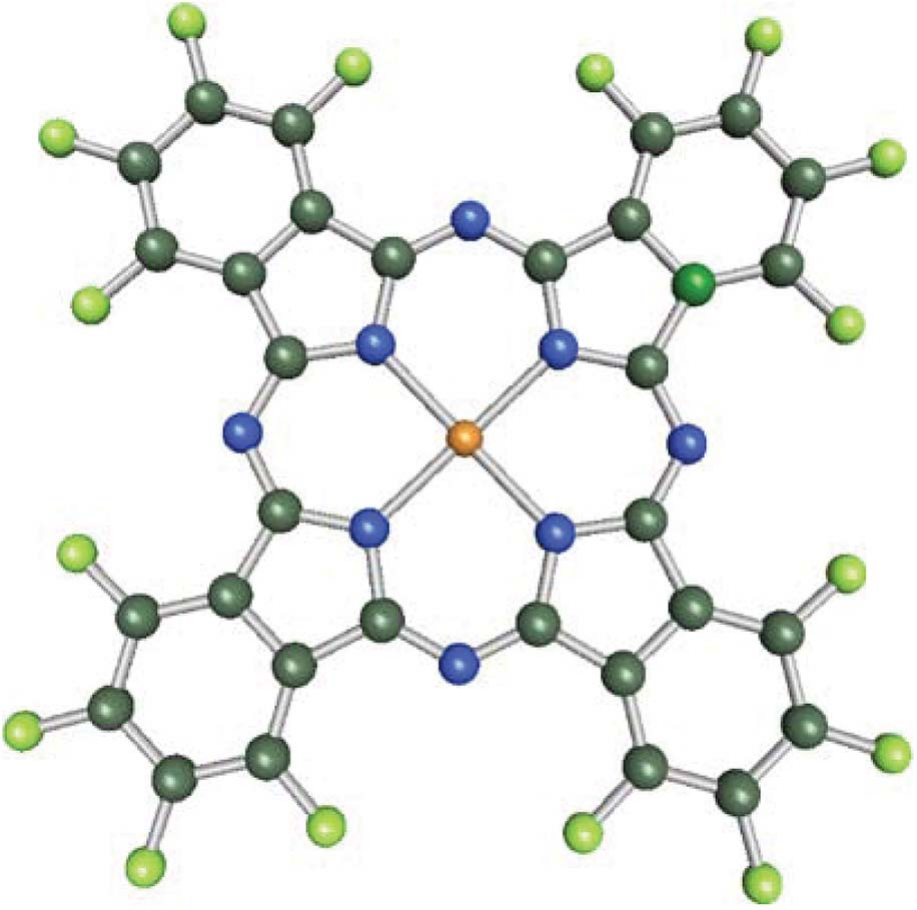
Schematic diagram of the F_16_MPcs molecules used in the present study.

Fig. 1 shows the schematic diagram of the F_16_MPcs molecules used in this work. Further, due to molecular functionalization, the molecular orbitals come closer to the Fermi level, leading to an increase in the ionization potential and electron affinity, which result in the preferred acceptor behaviour.^34^

## 2. Experimental

The SWCNTs and F_16_MPcs (where M = Co, Zn) samples were commercially procured from Sigma-Aldrich. The acidification of SWCNTs consisting of the acidic group (–COOH) was performed through the established multi-step acid treatment procedures.^35^ It is worth mentioning that the carboxyl group imparts negative charges and results in the long-term stability of the CNTs dispersion.^35,36^ Varying amounts of F_16_MPcs (0.1 to 0.5 wt%) were dissolved in 5 mL of dimethylformamide (DMF), and subsequently subjected to stirring to give the F_16_MPcs/DMF solution. The above solution was then successively and cautiously added dropwise to the SWCNTs–COOH (30 mg) suspensions in DMF and then sonicated at room temperature (25 °C) for 3 h and stirred in the dark for 6 h at 100 °C. After stirring and filtration through a PTEF filter (0.22 μm, Millipore), the product was washed thoroughly with DMF to eradicate the excess F_16_MPcs derivative, followed by washing with ethanol numerous times, then finally drying to acquire F_16_CoPc/SWCNTs–COOH (S_1_) and F_16_ZnPc/SWCNTs–COOH (S_2_) hybrids.

Raman spectra were obtained using a Renishaw inVia micro-Raman spectrometer. Fourier transform infrared (FT-IR) and ultraviolet-visible (UV-vis) spectra of S_1_ and S_2_ hybrids were obtained on a Perkin Elmer Frontier FT-IR spectrometer and UV-2450PC (Shimadzu, Japan) spectrophotometer, respectively. The morphologies of S_1_ and S_2_ hybrids were determined by field emission scanning electron microscopy (FE-SEM, Carl Zeiss, supra 55) and transmission electron microscopy (Jeol, TEM-2100). Thermogravimetric analysis (TGA) was performed using a thermogravimetric analyzer (Hitachi STA 7200) under a nitrogen atmosphere from 40 to 900 °C at a scan rate of 10 °C min^−1^. X-ray photoelectron spectroscopy (XPS) was conducted using a Mg K_α_ X-ray beam as the excitation source (1253.6 eV) and a MAC2 electron analyzer system attached to an MBE machine (EVA-32 Riber, France). The binding energy scale was calibrated to the Au 4f_7/2_ line of 84.0 eV.

**Fig. 2:**
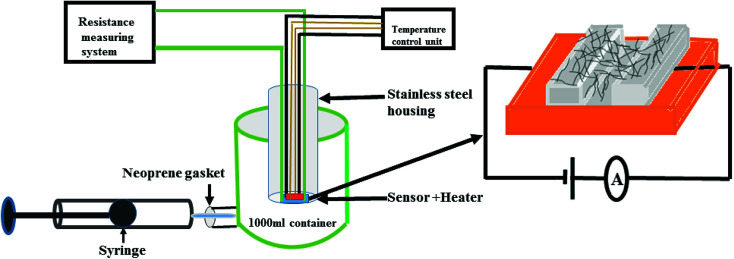
The gas sensing set-up used in the present study.

The gas sensing studies were carried out using a homemade gas handling test chamber (1000 mL) containing a sample holder geometry as shown in Fig. 2. To prepare gas sensors of S_1_ and S_2_ hybrids, 2 mg of the as-prepared F_16_MPcs/SWCNTs hybrids were dispersed in 1 mL of DMF and then multiple sensors with effective area of 3 mm × 1 mm were fabricated by drop casting 30 μL of hybrid solution onto a glass substrate with two precoated gold electrodes (3 mm × 3 mm at a spacing of 1 mm). Silver wires were attached to the gold electrodes using silver paste. Sensor resistance was recorded continuously by applying a constant bias of 3 V during both dosing and purging cycles as a function of time, using a computer interfaced Keithley electrometer 6517A. The desired concentrations of (NO_2_, NO, Cl_2_, H_2_S, C_2_H_5_OH, CO and NH_3_) gases in the test chamber were achieved by injecting a known quantity of gas using a micro-syringe; once steady-state was achieved after exposure, sensor resistance was recovered by opening the lid of the test chamber.

The response of the gas sensor was calculated using eqn (1):

1 *S*(%) = |(*R*_a_ − *R*_g_)/*R*_a_| × 100

where *R*_a_ and *R*_g_ represent the sensor resistance in air and gas environments, respectively. The response time is defined as the time needed for the sensor resistance to reach 90% of its final value after the gas is introduced into the test chamber, and recovery time is defined as the time required for the sensor resistance to regain 90% of its original value after the purging out of the gas. XPS studies of exposed samples were conducted by the *ex situ* exposure of gases to the samples in the gas sensing set up (Fig. 2) and transferring them to the XPS analysis chamber. The saturated aqueous solutions of LiCl, MgCl_2_, K_2_CO_3_, NaBr, KI, NaCl, KCl and K_2_So_4_ at an ambient temperature of 25 °C were used for maintaining homogeneous and stable environments with relative humidities of nearly 11.5%, 32.6%, 43.4%, 57.3%, 68.4%, 75.7%, 84.5% and 97.1 respectively.^37^ A hygrometer (Keithley 6517 A) was used to independently monitor the relative humidity (RH) levels. Impedance spectroscopy studies of the S_1_ and S_2_ hybrids were carried out using a frequency response analyser (FRA) attached with a potentiostat (Autolab) in the frequency range of 10 Hz to 1 MHz. In accordance with the sensing study, 0.3 wt% of S_1_ and S_2_ hybrid sensors were chosen for further study.

## 3. Results and discussion

### 3.1 Material characterization of the F_16_MPc/SWCNTs–COOH hybrid

**Fig. 3:**
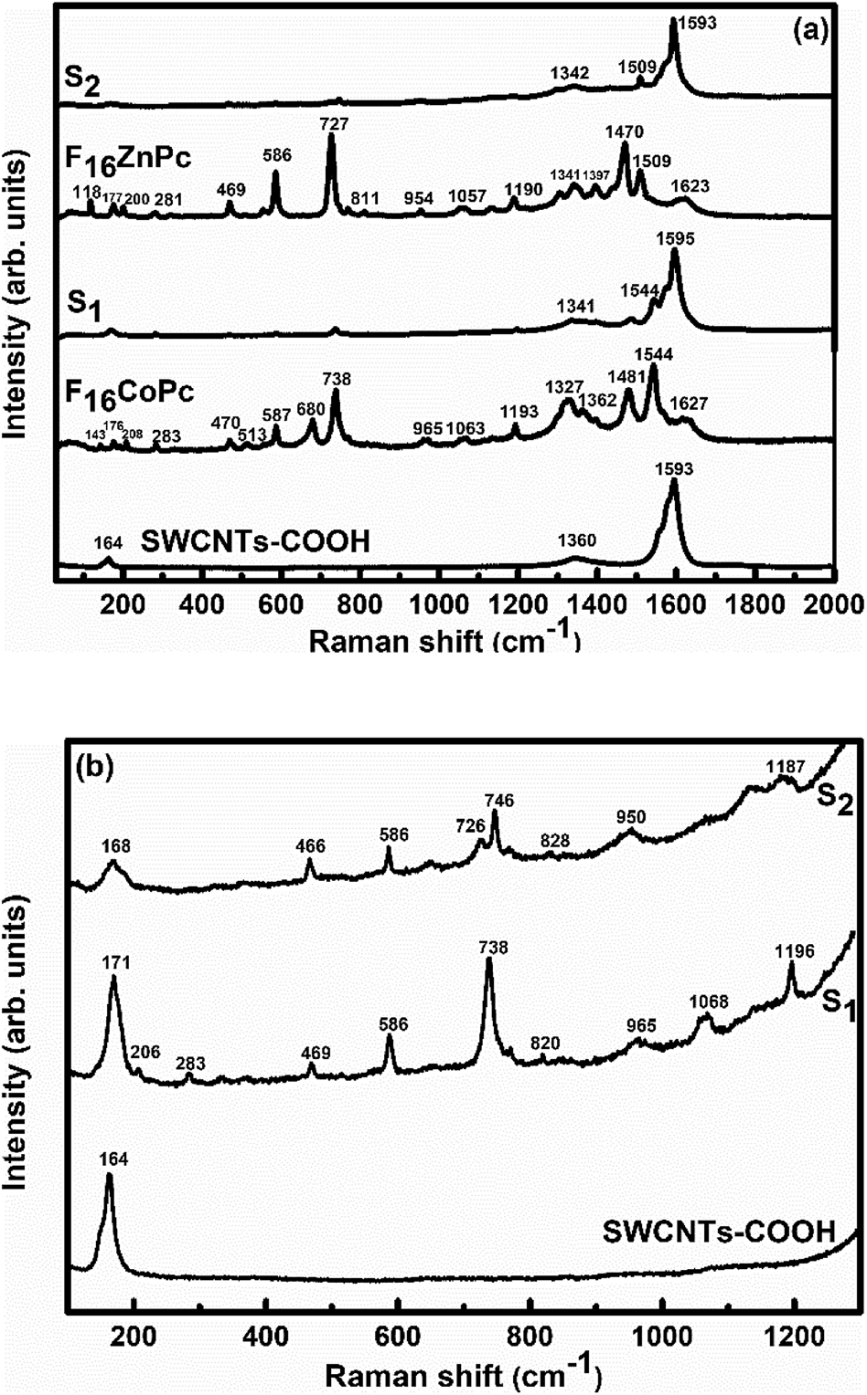
Raman spectra of (a) SWCNTs–COOH, F_16_CoPc, S_1_, F_16_ZnPc and S_2_ hybrids, and (b) SWCNTs–COOH, S_1_ and S_2_ hybrids in the 30–1300 cm^−1^ range (magnified view).

In order to explore the interactions between phthalocyanine molecules and SWCNTs–COOH, Raman and FTIR spectroscopic measurements of all the samples were conducted. The Raman spectra (Fig. 3) of SWCNTs–COOH contain the characteristic G-band due to the bond stretching of sp^2^ atoms at around 1593 cm^−1^ and the D band at around 1360 cm^−1^ due to the breathing mode of sp^2^ atoms.^38,39^ Moreover, a characteristic peak at 164 cm^−1^ is for the radial breathing mode (RBM) of SWCNTs–COOH, which signifies the distribution of diameters in the SWCNTs–COOH sample.^40^ The peaks at 143, 176, 208, 283, 470, 513, 587, 680, 738 and 965 cm^−1^ in F_16_CoPc and peaks at 118, 177, 200, 281, 469, 586, 727, 811 and 954 cm^−1^ in F_16_ZnPc are due to the vibrations of isoindole moieties.^41^ The characteristic peaks between 1200 and 1600 cm^−1^ are due to pyrrole groups. Moreover, bands at 1544 and 1509 cm^−1^ correspond to cobalt and zinc ions, in agreement with earlier studies.^41–43^ It is worth noting that a combination of peaks of both the F_16_MPcs and SWCNTs–COOH were found in the Raman spectra of S_1_ and S_2_ hybrids. Moreover, D and G bands were found to be marginally broadened due to the superimposition with F_16_MPc peaks. Fig. 3(b) shows enlarged parts of the spectra from 100 to 1300 cm^−1^, in which there is a change in the peak position and intensity of the characteristic Raman peak of the phthalocyanine macrocycle by interaction with SWCNTs–COOH. The relative intensity ratio (*I*_D_/*I*_G_) was determined to be 0.3, 0.19 and 0.24 for SWCNTs–COOH, S_1_ and S_2_ samples, respectively.^28,40^ A small variation in *I*_D_/*I*_G_ demonstrated that F_16_MPc molecules are non-covalently attached to the surface of SWCNTs–COOH.^39,40^ Nevertheless, π–π stacking interactions between SWCNTs–COOH and F_16_MPcs aromatic rings resulted in the shift of RBM towards a higher frequency.^44,45^ The higher frequency shift in the S_1_ hybrid as compared to the S_2_ hybrid indicates that the adsorption of F_16_CoPc induces a more significant shift in comparison to F_16_ZnPc, due to the enhanced F_16_CoPc molecule-SWCNT–COOH interaction.^44^

**Fig. 4:**
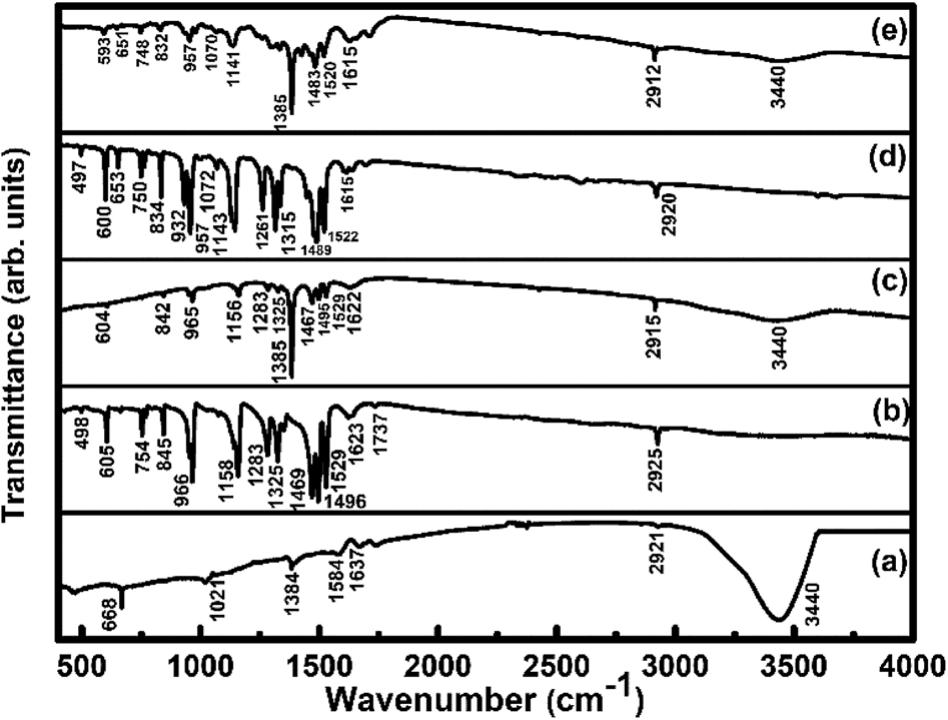
FTIR spectra of (a) SWCNTs–COOH; (b) F_16_CoPc; (c) S_1_; (d) F_16_ZnPc and (e) S_2_ hybrid.

FTIR spectra (Fig. 4) of SWCNTs–COOH show the C–O stretching vibration peak at 1021 cm^−1^, the O–H stretching vibration peak at 3440 cm^−1^ due to the carboxylic group^36^ and a peak at 1637 cm^−1^ due to the C=C stretching vibration.^36^ The peaks at 2855 and 2921 cm^−1^ correspond to asymmetric and symmetric CH_2_ stretching vibrations.^46^ The observed IR peaks at 498, 605, 754, 845, 966, 1158 cm^−1^ for the F_16_CoPc sample and at 497, 600, 653, 750, 834, 932, 957, 1072, 1143 cm^−1^ in the F_16_ZnPc sample are due to the hexadecafluoro substituents. The presence of other peaks at 1283, 1325, 1469, 1496, 1529, 1623, 1737, 2925 cm^−1^ for F_16_CoPc and at 1261, 1315, 1489, 1522, 1615, 2920 cm^−1^ for F_16_ZnPc are due to aliphatic C–H vibrations.^20^ The peaks appearing in F_16_CoPc and SWCNTs–COOH can be found in the S_1_ hybrid at 604, 842, 965, 1156, 1283, 1325, 1385, 1467, 1495, 1529, 1622 and 2915 cm^−1^.^47^ Similarly, peaks appearing in F_16_ZnPc and SWCNTs-COOH are observed in the S_2_ hybrid at 593, 651, 748, 832, 957, 1070, 1141, 1385, 1483, 1520, 1615 and 2912 cm^−1^.^47^ It is worth mentioning that the peak appearing at 1385 cm^−1^ in both the S_1_ and S_2_ hybrids is due to the C–N–C vibration,^48^ which confirms the interaction between phthalocyanine and CNTs. The characteristic peaks of the phthalocyanine molecule in the IR spectra of S_1_ and S_2_ hybrids are found to be slightly red shifted in wavenumber in comparison to their individual peaks. Nevertheless, a higher shift in wavenumber in the S_1_ hybrid in comparison to S_2_ reveals that adsorption of F_16_CoPc induces a more significant shift due to the enhanced electron delocalization *via* π–π stacking interactions between the F_16_CoPc molecule and SWCNT–COOH, which is concomitant with Raman spectroscopic studies.^49^

**Fig. 5:**
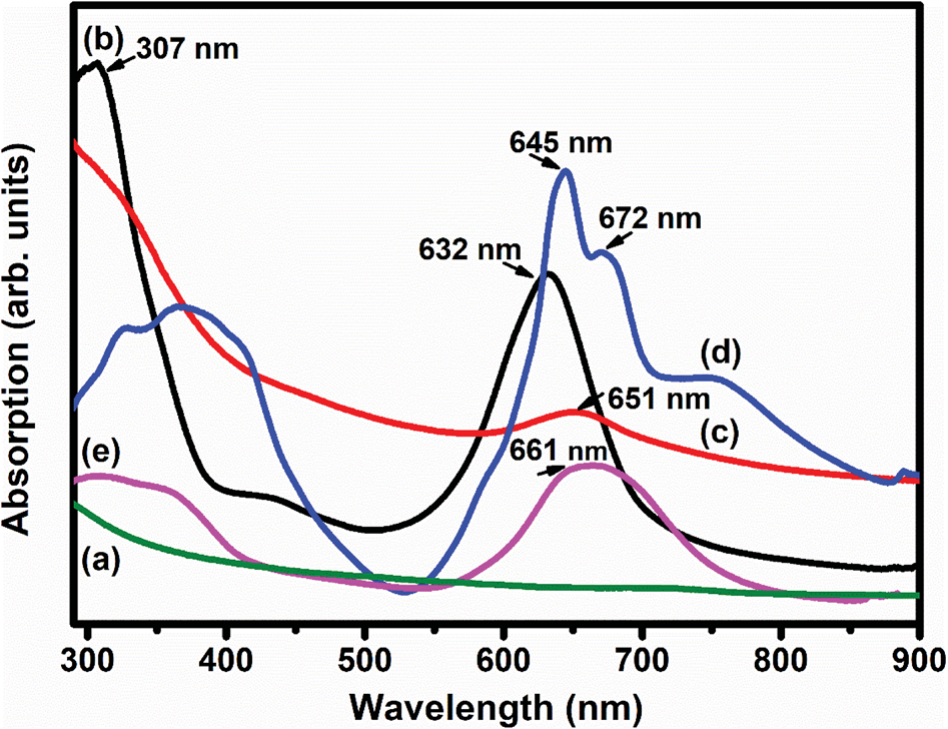
UV-vis absorption spectra of (a) SWCNTs–COOH; (b) F_16_CoPc; (c) S_1_; (d) F_16_ZnPc and (e) the S_2_ hybrid.

Fig. 5 depicts the UV-vis absorption spectra of SWCNTs–COOH, F_16_MPcs, S_1_ and S_2_ hybrids. The UV-vis absorption spectrum of SWCNTs–COOH was observed to be featureless^50^ and the spectra of F_16_MPcs exhibit two strong absorption bands, one broad B band in the wavelength range 305–371 nm due to the electronic transitions from the HOMO a_2_u to the LUMO e_g_ level and the Q band in the visible range at 632–672 nm is because of the electronic transitions from the HOMO a_1_u level to the LUMO e_g_ level.^51^ However, in the case of S_1_ and S_2_ hybrids, the Q-band was found to be comparatively broadened with a decrease in absorption in the dispersions containing F_16_MPc/SWCNTs–COOH and their maxima were red-shifted by 19 and 16 nm, respectively, as compared to that of the individual F_16_MPc spectrum. It is worth mentioning that the expanded macrocyclic conjugated structure of F_16_MPc and the reduced energy difference between the HOMO and the LUMO facilitates charge transfer between F_16_MPc and SWCNTs–COOH. In addition, the higher red-shift in the S_1_ hybrid as compared to S_2_ confirms the significant π–π interaction and the charge transfer between F_16_CoPc and SWCNTs–COOH.^40^

**Fig. 6:**
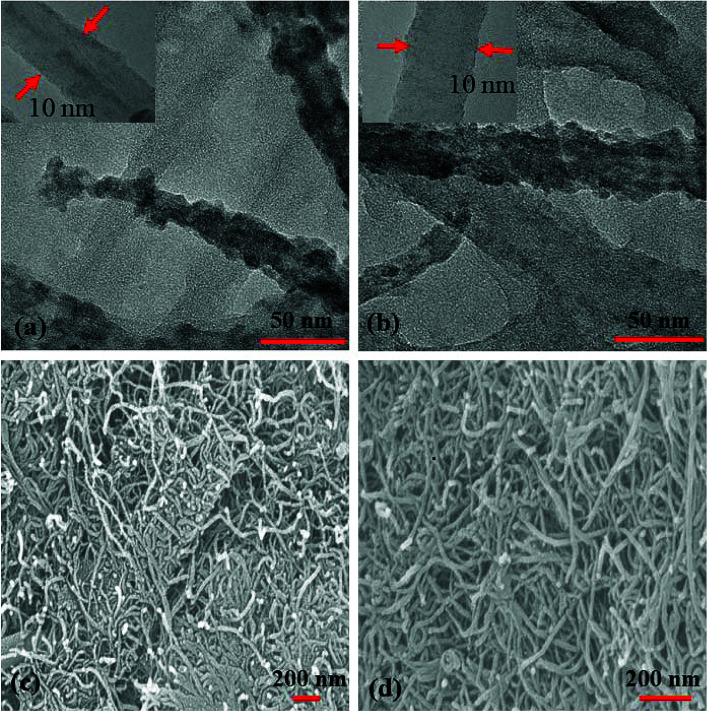
(a and b) TEM images (insets show the TEM images of SWCNTs–COOH) and (c and d) SEM images of the S_1_ and S_2_ hybrids.

**Fig. 7:**
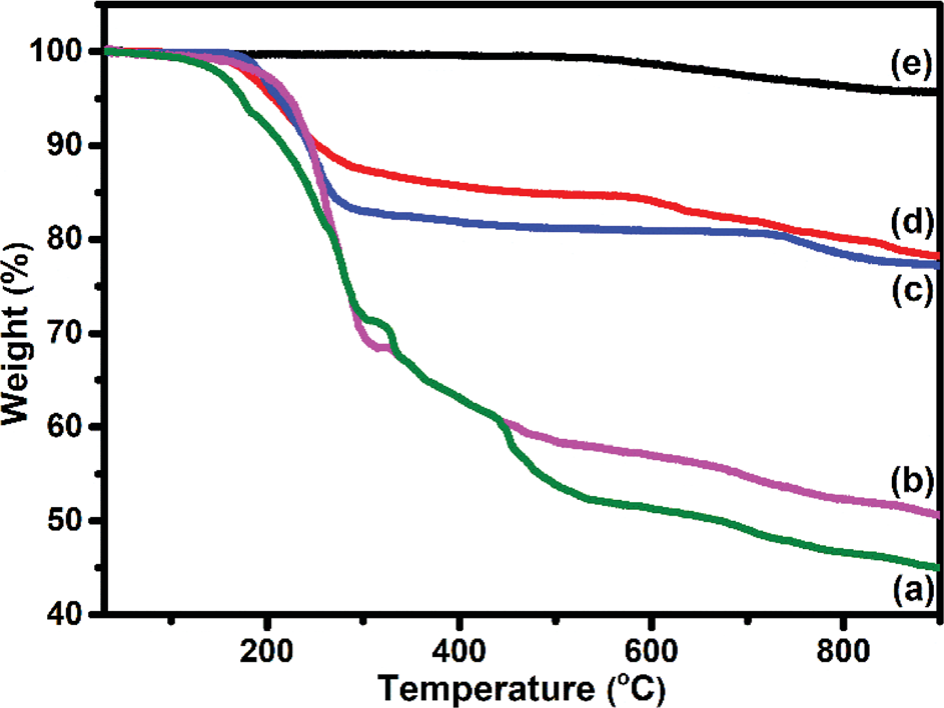
TGA curves of (a) F_16_ZnPC, (b) F_16_CoPC, (c) S_2_ (d) S_1_ hybrid and (e) SWCNTs–COOH.

TEM (Fig. 6(a and b)) images of S_1_ and S_2_ hybrids demonstrate the exohedral anchoring of phthalocyanine molecules on the walls of SWCNTs–COOH with a mean diameter of about 36 and 29 nm in comparison to SWCNTs–COOH (inset view) with diameter of about 10 nm. Additionally, scanning electron microscopy images (Fig. 6(c and d)) of S_1_ and S_2_ hybrids also highlight that phthalocyanine molecules are anchored on the surface of the SWCNTs–COOH matrix, making a thicker SWCNTs–COOH surface in contrast to individual SWCNTs–COOH. The weight loss as a function of temperature for SWCNTs–COOH, F_16_ZnPc, F_16_CoPc, S_1_ and S_2_ hybrid materials has been investigated using TGA plots (Fig. 7)).

A loss of weight of about 54.60% and 49.05% up to 900 °C was observed for F_16_ZnPc and F_16_CoPc (Fig. 7(a and b)), comprised of major weight losses in steps from 200 to 330 °C and 366 to 604 °C due to the desorption of adsorbed water and the decomposition of F_16_MPc, respectively.^45,52^ TGA plots of SWCNTs–COOH (Fig. 7(e)) exhibited a weight loss of about 4.21% due to the destruction of the residual carbon and decarboxylation of oxidized species.^40^ In contrast, S_1_ and S_2_ (Fig. 7(c and d)) had weight losses of 21.83% and 22.79%, respectively, on heating the hybrid to 900 °C, corresponding to the decomposition of the F_16_MPc on the SWCNTs–COOH surface.^45^ The amount of F_16_MPc molecules adsorbed on the SWCNTs–COOH was calculated using the ratio of the difference in weight loss between SWCNTs–COOH and the F_16_MPc/SWCNTs–COOH hybrid to the weight loss for F_16_MPc and was found to be 35.92% and 34.02% for the S_1_ and S_2_ hybrids, respectively.

### 3.2 Gas sensing measurements

**Fig. 8:**
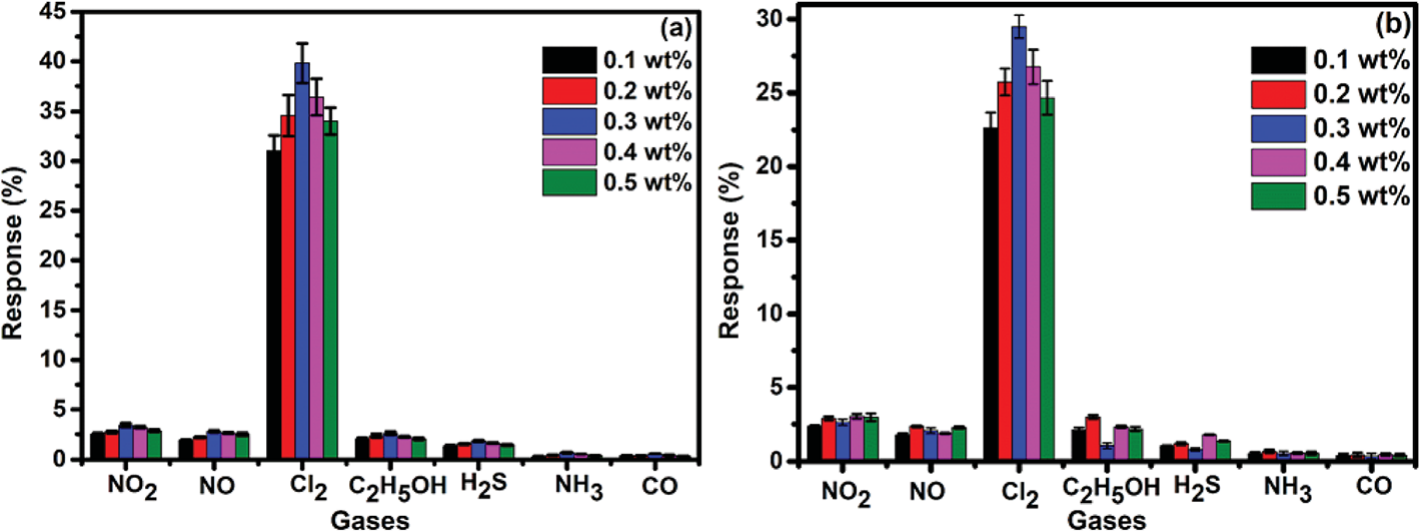
(a and b) Selectivity histogram of S_1_ and S_2_ sensors for 500 ppb of NO_2_, NO, Cl_2_, C_2_H_5_OH, H_2_S, NH_3_ and CO at room temperature.

**Fig. 9:**
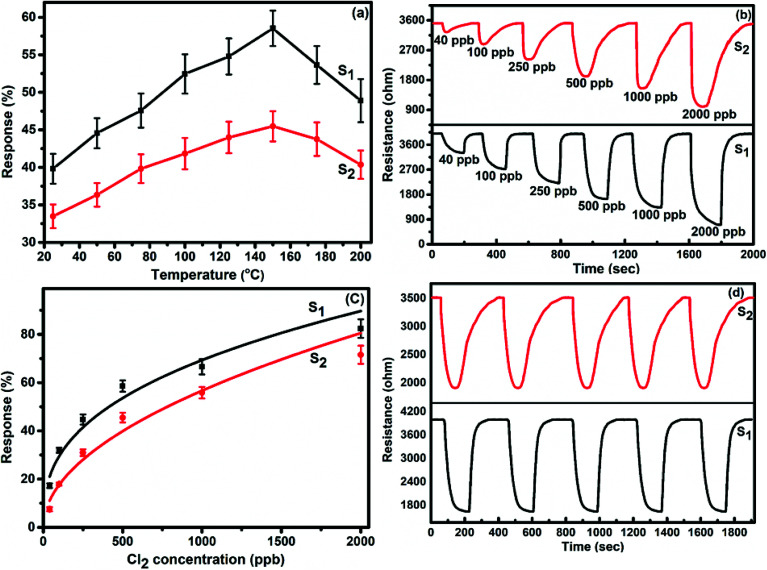
(a) The response of S_1_ and S_2_ sensors as a function of temperature to 500 ppb of Cl_2_. (b) Response curves of S_1_ and S_2_ sensors for different doses of Cl_2_ at 150 °C. (c) Variation in the response of S_1_ and S_2_ sensors with Cl_2_ concentration (experimental curve (dotted lines) and the fitting curve (solid lines)). (d) Reproducibility of the response curve of the S_1_ and S_2_ sensor to 500 ppb of Cl_2_ at 150 °C. (The standard error bars indicate the response variations after testing three sensor devices for three times).

To demonstrate the gas sensing properties of prepared S_1_ and S_2_ hybrid sensors, we recorded the response curves (change in resistance of the film as a function of time) of the sensors to 500 ppb of different test gases at room temperature (25 °C). From the selectivity histogram (Fig. 8), it can be seen that for the tested gases at room temperature, 0.3 wt% of S_1_ and S_2_ hybrid sensors exhibited the best response towards Cl_2_ among all the prepared sensors, with sensitivity values of ∼40% and 30%, respectively, in comparison to the pristine SWCNTs sensor with a sensitivity value of ∼1%.^28^ This indicates that the F_16_MPcs molecule enhances the sensor response because of the synergic interaction of MPcs with CNTs due to π–π interactions. As such, 0.3 wt% of S_1_ and S_2_ hybrid sensors was chosen for further sensing studies. The sensitivity values for all other tested gases were <4%. Moreover, at room temperature, the sensors showed irreversible behaviour, as they did not recover to the baseline resistance even after a long interval of time. It was observed that heating improves the recovery characteristics of the sensors; the operating temperature was optimized in order to make the sensors reversible. Here, both sensors S_1_ and S_2_ were exposed to 500 ppb of Cl_2_ at different operating temperatures ranging from 25 °C to 200 °C. A plot of sensor response for 500 ppb of Cl_2_ as a function of temperature is shown in Fig. 9(a). The response for Cl_2_ was rapidly enhanced with increasing temperature and the maximum responses of ∼59% and 46% were obtained for sensors S_1_ and S_2_, respectively, at 150 °C. Furthermore, the response of the sensors decreases beyond 150 °C, due to desorption of Cl_2_ from the surface of the sensors.

Fig. 9(b) shows the resistance variation in S_1_ and S_2_ sensors as a function of time for different concentrations of Cl_2_ (40–2000 ppb) at 150 °C. Upon exposure to Cl_2_, the sensor resistance decreases and it becomes saturated after some time; after purging with air, it again starts approaching its initial baseline value, indicating good reversibility. Fig. 9(c) demonstrates the response behaviour of S_1_ and S_2_ sensors to 40–2000 ppb concentrations of Cl_2_. The response value of the sensors increased with increasing the concentration of Cl_2_. The responses of S_1_ and S_2_ sensors were found to lie in the range of ∼18–82% and ∼8–72%, respectively. It is worth mentioning that the response of F_16_MPc/SWCNTs–COOH hybrids towards chlorine is greater than that of F_16_MPc/MWCNTs–COOH hybrids.^28,29^ Further, due to certain superior features of SWCNTs such as smaller size, stronger inter-tube attraction and larger specific surface area compared to the MWCNTs, the gas sensing parameters of the SWCNTs-based sensors are enhanced. The response of SWCNTs hybrids decreases in the order F_16_CoPc > F_16_ZnPc > F_16_CuPc,^28^ which has been clarified in terms of the central ion size; *i.e.*, the larger ionic radius and especially the interaction effects between Cl_2_ and different central ions. It was found that the interactions increase with the corresponding increase in the atomic size of the atoms/ions for a given separation distance because larger atoms are more easily polarizable and provide more electrons to polarize, which results in strong van der Waals forces, indicating that the central metal size plays an important role in the sensitivity of Cl_2._ This is in agreement with charge transfer and the number of Pc molecules adsorbed onto the SWCNTs wall, as estimated by TGA and Cl_2_ interactions with the sensor as observed in X-ray photoelectron and EIS studies discussed in Section 3.3.^53^Fig. 9(d) shows the response curves of S_1_ and S_2_ sensors for successive exposures to Cl_2_. The sensors showed no significant changes in response and recovery characteristics after repeated gas exposure, demonstrating the reproducible and stable sensing characteristics of the sensors.

**Fig. 10:**
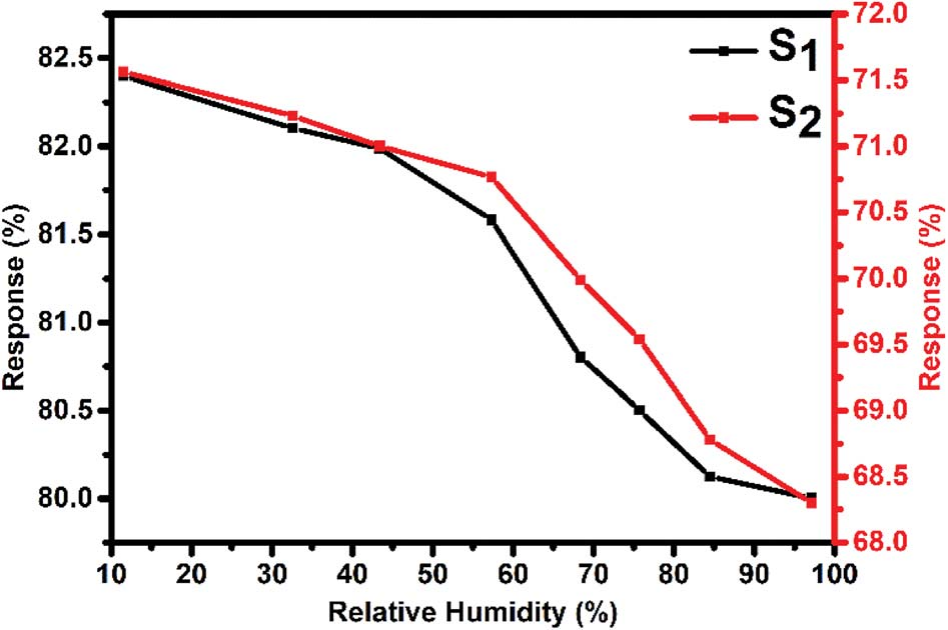
Variation in the Cl_2_ response of S_1_ and S_2_ sensors with humidity for 2 ppm of Cl_2_ at room temperature.

Fig. 10 represents the variation in the Cl_2_ response of S_1_ and S_2_ sensors with relative humidity (11–98%) for 2000 ppb of Cl_2_ at room temperature. It was observed that both sensors showed only small variations (2.4% for S_1_ and 3.26% for S_2_ sensor) in their Cl_2_ response as the humidity level was varied from 11% to 98%, indicating that humidity has a negligible effect on the Cl_2_ response of these sensors.

The response variation with the gas concentration was studied using eqn (2):^54,55^2
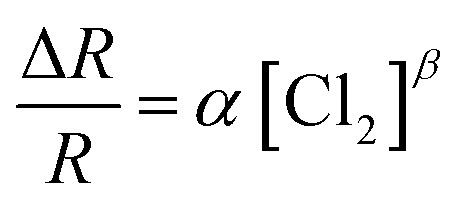
where *α* is the coefficient for the adsorption capacity and *β* is the strength of adsorption,^55^ which are obtained by curve fitting of the response curve. It is interesting to note that a smaller value of *β*, lying between 0 and 1 for normal adsorption, signifies the greater heterogeneity of the sample. The values of *α* and *β* were calculated to be 6.94 and 0.32 for S_1_ and 2.62 and 0.43 for S_2_ hybrids. A *β* value less than one indicates the normal mode of adsorption on the heterogeneous surface of the sensor.^54^

In the above experimental study, the lowest detectable concentration is limited due to the experimental set up used. Nevertheless, the limit of detection (LOD) of the sensor was derived from the signal-to-noise ratio (S/N), which is defined as Δ*R*/*σ*, where Δ*R* is the maximum resistance change with respect to *R*_a_ (baseline resistance) and *σ* represents the root mean square (rms) noise of the baseline in air.^56^

The LOD was calculated by using the eqn (3):^57^3
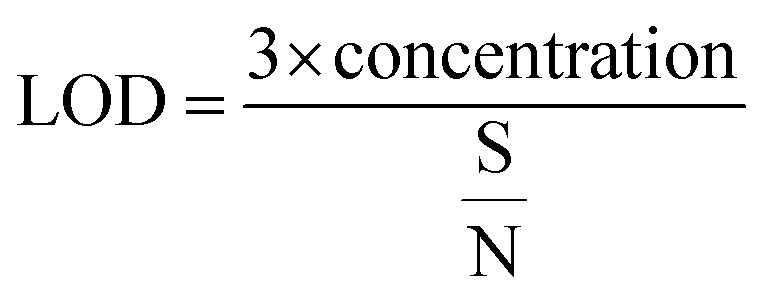


The signal-to-noise ratios of sensors S_1_ and S_2_ are 3000 and 2400, respectively, with the corresponding detection limits of 0.04 ppb and 0.05 ppb, respectively. Thus, the higher sensitivity, reversibility and reproducibility of the F_16_MPc/SWCNTs hybrid sensor in comparison to other CNTs-based Cl_2_ sensors reported in literature^1,58^ make these sensors favourable candidates for ppb level Cl_2_ detection.

### 3.3 Gas sensing mechanism

**Fig. 11:**
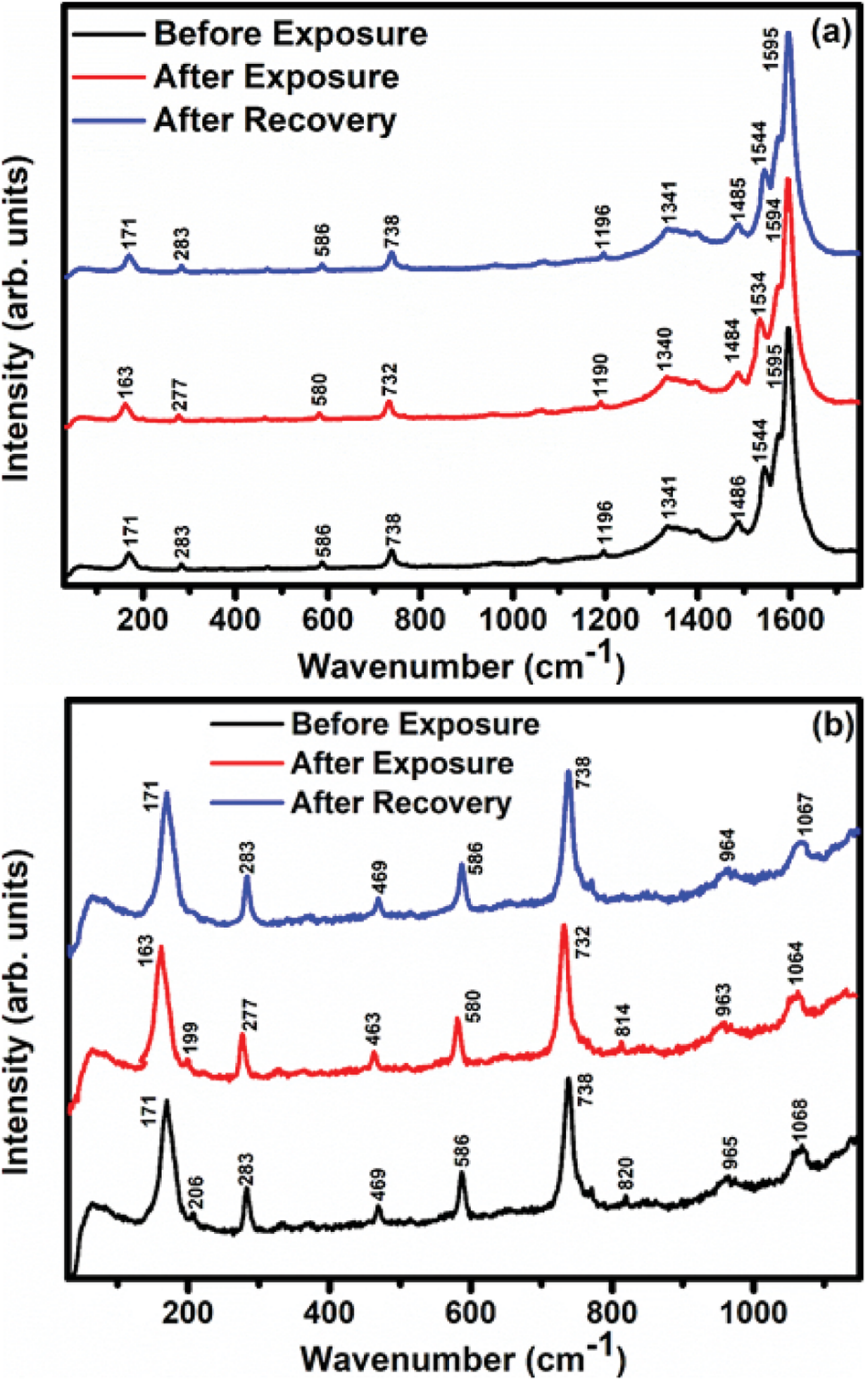
Raman spectra of the S_1_ sensor recorded (a) before exposure (black curve), after exposure (red curve) to 25 ppm of Cl_2_ and after full recovery (blue curve); (b) magnified view in the range 30–1300 cm^−1^.

**Fig. 12:**
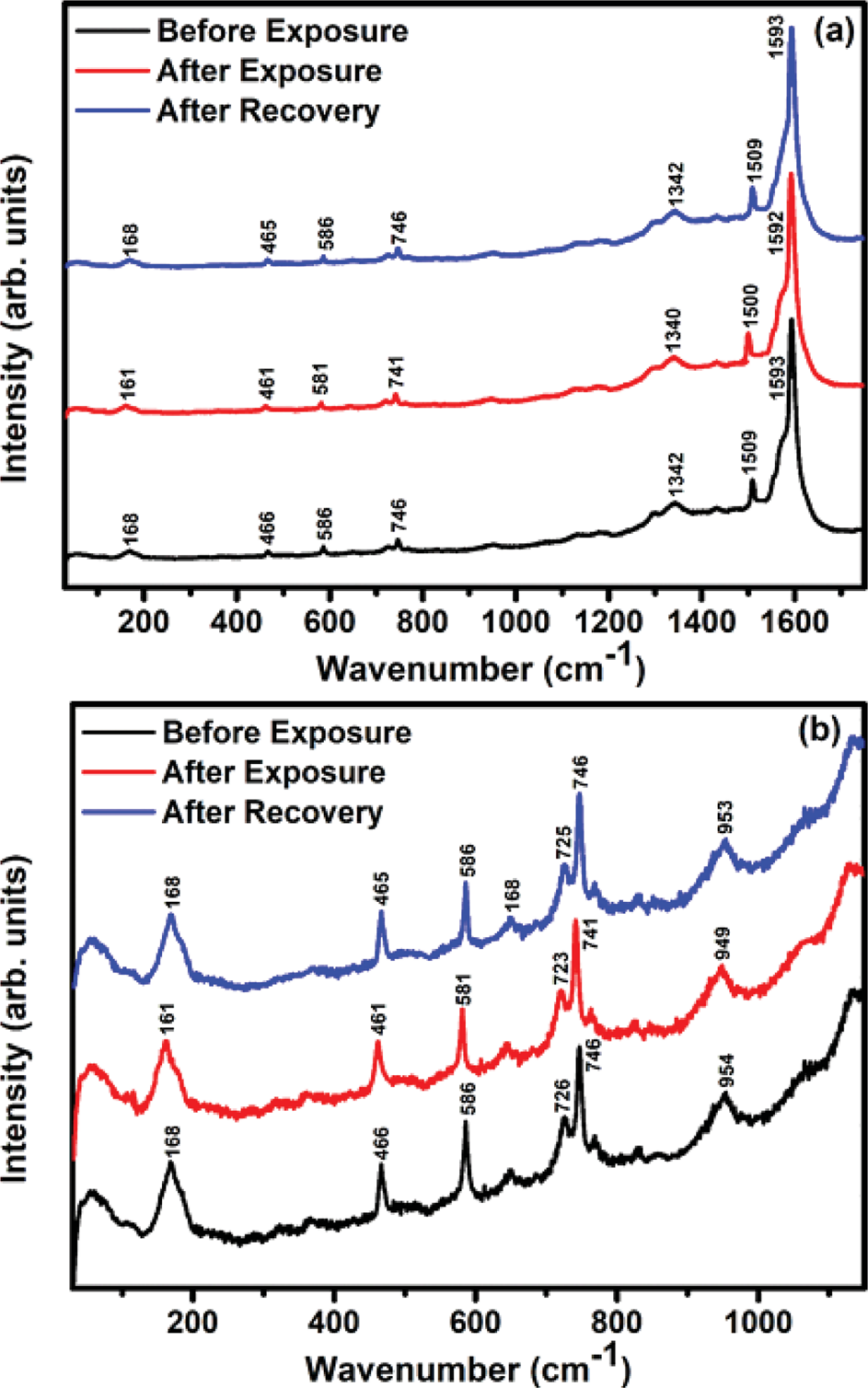
Raman spectra of the S_2_ sensor recorded (a) before exposure (black curve); after exposure (red curve) to 25 ppm of Cl_2_ and after full recovery (blue curve) (b) in the range 30–1300 cm^−1^ (magnified view).

Raman, XPS and impedance spectroscopic measurements of S_1_ and S_2_ hybrid sensors have been performed both in air and after purging in Cl_2_ in order to explore the sensing mechanism of the sensors. On exposure of the S_1_ sensor to Cl_2_, the Raman peak (Fig. 11) corresponding to the cobalt–nitrogen bond^59^ (171 cm^−1^) is shifted by 8 cm^−1^ and macro-cyclic vibration^60^ peaks (283, 586, 738, 820, 1196 cm^−1^) are shifted by 6 cm^−1^, whereas D and G bands corresponding to SWCNTs–COOH (1341 and 1595 cm^−1^) are shifted by 1 cm^−1^.^38^ In contrast, in the Raman spectra (Fig. 12) of sensor S_2_, the peak corresponding to the zinc–nitrogen bond^59^ (168 cm^−1^) is shifted by 7 cm^−1^ and macro-cyclic vibration^60^ peaks (119, 466, 586, 746 cm^−1^) are shifted by 5 cm^−1^, whereas D and G bands corresponding to SWCNTs–COOH (1342 and 1593 cm^−1^) are shifted by 2 and 1 cm^−1^, respectively.^38^ The major shift of 10 cm^−1^ in S_1_ and 9 cm^−1^ in S_2_ (1544 cm^−1^ and 1509 cm^−1^) corresponds to the displacement of the C–N–C bridge bond, closely linked to the cobalt and zinc ions of the phthalocyanine molecule.^43^ The higher shift in the S_1_ hybrid as compared to the S_2_ hybrid indicates the predominant interaction of Cl_2_ with the cobalt ions of the hybrid sensor. The Raman spectra of the hybrid sensors recorded after purging Cl_2_ showed identical peaks to those of fresh samples, which reflects the excellent reversibility of these sensors.

**Fig. 13:**
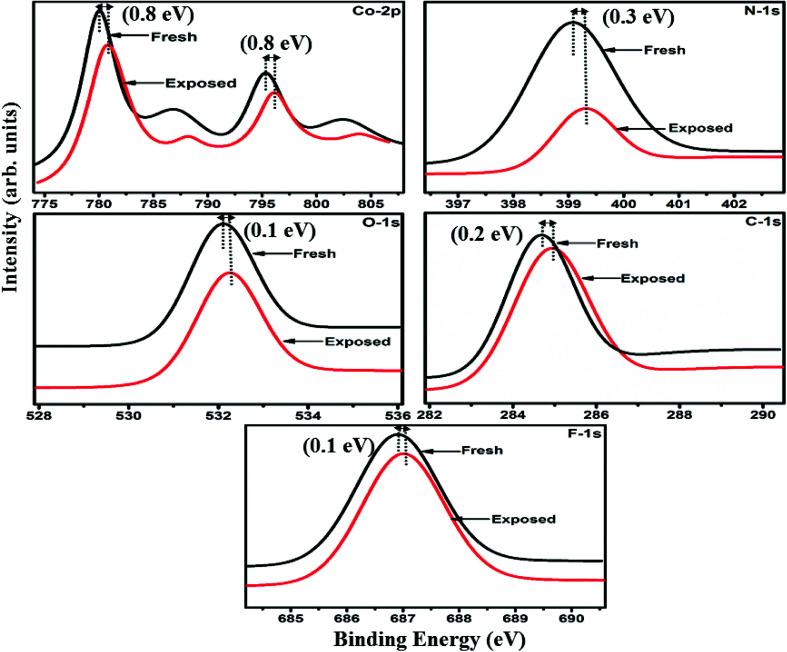
XPS spectra of the S_1_ sensor recorded before and after exposure to 25 ppm of Cl_2_.

**Fig. 14:**
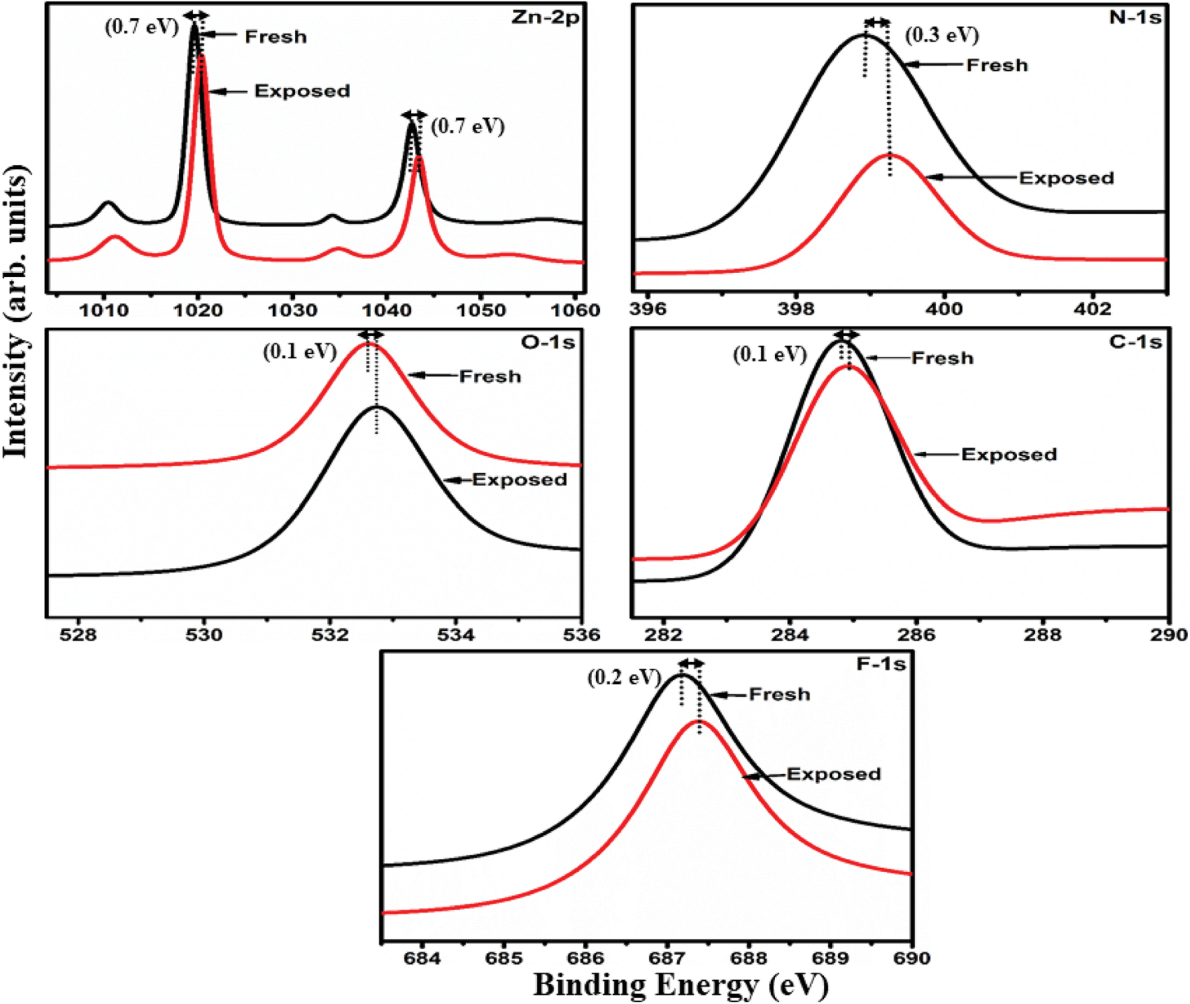
XPS spectra of the S_2_ sensor recorded before and after exposure to 25 ppm of Cl_2_.

The interactions between Cl_2_ and the S_1_/S_2_ hybrid sensors were further confirmed by observing the shifts in binding energy in the XPS spectra of unexposed and Cl_2_ exposed samples. The XPS spectrum (Fig. 13) of the fresh S_1_ hybrid shows characteristic peaks at 284.7, 532.1, 399.0, 686.9, 780.0, 795.3 eV corresponding to C-1s, O-1s, N-1s, F-1s, Co-2p_3/2_ and Co-2p_1/2_ levels, respectively.^40^ Once the sample was exposed to Cl_2,_ there was a peak shift of 0.2 eV in the spectrum of the core level C-1s, a shift of 0.1 eV in the spectrum of O-1s and F-1s, a shift of 0.3 eV in the spectrum of N-1s and a prominent peak shift of 0.8 eV in the core level spectrum of Co-2p. In contrast, the XPS spectrum (Fig. 14) of the fresh S_2_ hybrid showed characteristic peaks at 284.8, 532.6, 399.0, 687.1, 1019.6, 1042.6 eV corresponding to C-1s, O-1s, N-1s, F-1s, Zn-2p_3/2_ and Zn-2p_1/2_ levels^24^ and after Cl_2_ exposure, there was a peak shift of 0.2 eV in spectrum of core level F-1s, a shift of 0.1 eV in the spectra of C-1s and O-1s, a shift of 0.3 eV in the spectrum of N-1s and a prominent peak shift of 0.7 eV in the core level spectrum of Zn-2p. The prominent shift of 0.8 eV towards the higher BE side in the Co-2p core level in the S_1_ hybrid, a shift of 0.7 eV towards the higher BE side in the Zn-2p core level in the S_2_ hybrid and a shift of 0.5 eV towards the higher BE side in Cu-2p^28^ confirm that charge transfer interactions occur upon adsorption of strong electron acceptor Cl_2_ molecules to the hybrid, leading to a decrease in electron density due to the transfer of electrons from the hybrid to Cl_2_.^29,61^ Thus, the analysis of the Raman and XPS spectroscopic observations is concomitant with the higher sensing response of the S_1_ hybrid because greater charge transfer takes place between Cl_2_ and the S_1_ hybrid through the central metal ion with the adsorption of Cl_2_. Nevertheless, charge can favourably travel from CNTs to F_16_MPcs, which leads to an increase in the hole concentration in CNTs and results in the fast variation in resistance as observed in Fig. 9(b).^61,62^ It is worth mentioning that there was no shifting of peak position in the XPS spectrum after recovery, and the absence of any chlorine signal confirms that the sensing process is highly reproducible.

**Fig. 15:**
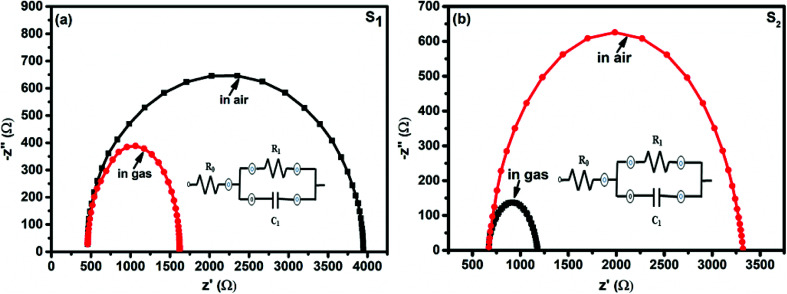
Impedance spectra of fresh and Cl_2_ exposed S_1_ and S_2_ sensors (the insets show the equivalent circuits used for the analysis of data obtained from the S_1_ and S_2_ sensors).

The interaction between the F_16_MPc/SWCNTs–COOH sensor and Cl_2_ has also been further studied using impedance spectroscopy tools, providing information about F_16_MPc/SWCNTs–COOH grains and the respective grain boundaries in accordance with morphological studies. Fig. 15(a and b) shows the impedance spectra of the F_16_MPc/SWCNTs–COOH sensor, obtained in air and under exposure to 500 ppb of Cl_2_, *i.e.*, the Cole–Cole plot.^53,63^ With an equivalent circuit (in the inset in Fig. 15) consisting of the RC network in series with a resistor *R*_0_, a single semi-circle was observed before and after exposure to Cl_2_. The intercept of the arc at high frequency with the real axis gives the grain resistance (*R*_0_). The resistance across the grain boundary (*R*_1_) was found from the diameter of the arc in Fig. 15, whereas the capacitance across the grain boundary (*C*_1_) was estimated from the relation.*ω*_max_*R*_1_*C*_1_ = 1,where *ω*_max_ is the frequency corresponding to the top of the arc.^53^ The equivalent circuit^63^ can be described as follows:

4 *Z* = *Z*′ + *jZ*′′

where *Z*′ = *R*_0_ + [*R*_1_/(1 + *ωR*_1_*C*_1_)^2^] and*Z*′′ = [*ωR*^2^_1_*C*_1_/(1 + *ωR*_1_*C*_1_)^2^].

Interestingly, the parameter *R*_0_ remained the same in S_1_ and S_2_ sensors in air and on exposure to Cl_2_, but *R*_1_ decreased and *C*_1_ increased in the presence of Cl_2_. This result is also supported by the fact that *R*_1_ changes across the grain boundary in the order of S_1_> S_2_> H_1_ (ref. 28) for hybrid sensors, indicating that incoming Cl_2_ molecules were adsorbed onto the outer surfaces of the grains and resulted in increased hole conductivity because of the charge transfer between phthalocyanines and CNTs, as explained in XPS investigations (Table 1).^28,53^

**Table 1:** Impedance parameters obtained for S_1_ and S_2_ sensors by fitting experimental data to the equivalent circuit

Sensors	Conditions	Parameters		
		*R*_0_ (Ω)	*R*_1_ (Ω)	*C*_1_ (nF)
S_1_	Unexposed	458	3488	2
	Exposed to 500 ppb Cl_2_	458	1164	5
S_2_	Unexposed	671	2649	3
	Exposed to 500 ppb Cl_2_	671	507	11

## 4. Conclusions

We have fabricated F_16_CoPc/SWCNTs–COOH and F_16_ZnPc/SWCNTs–COOH hybrid sensors using the solution assembly route, through π–π stacking interactions between F_16_MPc and SWCNTs–COOH for chlorine sensing applications. The results demonstrate that the F_16_CoPc/SWCNTs–COOH sensor exhibits high sensitivity (∼82% for 2 ppm with LOD of 0.04 ppb), excellent reproducibility and selectivity towards chlorine with response decreasing in the order of Co > Zn > Cu, indicating that the central metal ions play an important role in the sensitivity of Cl_2_, and this is in good agreement with the central ion size: the larger the ionic radius, the greater the charge transfer and Cl_2_ interaction with the sensor as observed from X-ray photoelectron, Raman and electrochemical impedance spectroscopic studies. Such effectiveness of the sensor originates from the synergetic interaction between F_16_MPc and SWCNTs–COOH. Strong response and good selectivity underline the significant potential of these hybrid materials in designing a new low-cost Cl_2_ sensor.

## Conflicts of interest

There are no conflicts to declare.

## Supplementary Material
